# Haplotyping via minimum recombinant paradigm

**DOI:** 10.1186/1753-6561-3-s1-s7

**Published:** 2009-02-23

**Authors:** Jules Hernández-Sánchez, Sara Knott

**Affiliations:** 1 Institute of Evolutionary Biology, University of Edinburgh, King's Buildings (Ashworth Laboratories), West Main Roads, EH9 3JT, Edinburgh, UK

## Abstract

**Background:**

Haplotypes can increase the power of gene detection over genotypes and are essential to estimate linkage disequilibrium.

**Methods:**

Haplotyping was based on the minimum recombinant paradigm, whereby a phase is obtained only if it uniquely minimises the number of recombinants within a full sib family. Performance of this method was tested across three different data sets, consisting of genotypes and pedigree.

**Results:**

The percentage of phased alleles ranged from ~80% to ~95%, and the percentage of correct phases reached ~99% in all cases. A measure of uncertainty was obtained via simulations. A partial haplotyping algorithm consisting of four deterministic rules was almost as effective as a full one consisting of six deterministic rules, and took up to 5 times less time to compute.

**Conclusion:**

Haplotyping via the minimum recombinant paradigm is consistently reliable and computationally efficient. A single simulation is enough to produce a population-wide uncertainty estimate associated with a set of all reconstructed haplotypes.

## Background

Using haplotypes can increase power of QTL mapping over genotypes, and are essential for estimating linkage disequilibrium. However, haplotypes are usually not available but must be obtained given genotypes and pedigree. The minimum recombinant haplotype configuration (MRHC) paradigm is a parsimonious method to obtain haplotypes given genotypes and pedigree [1]. Under the MRHC paradigm, one phase, or parental origin of alleles, is preferred over all other possible phases when it uniquely minimises the number of recombination events observed within a full sib family.

The MRHC, as implemented by Qian and Beckmann, consists of 6 rules. Rule 1 recovers missing alleles when certainty is absolute. Rule 2 phases alleles in progeny when certainty is absolute based on parental genotypes. Rule 3 phases alleles in founders given haplotypes in their progeny and MRHC. Rule 4 assigns phases to progeny given parental haplotypes and the MRHC paradigm. Rule 5 recovers additional missing alleles in parents given haplotypes in a full sib family and the MRHC paradigm. Rule 6 phases ambiguous loci that have not been phased by rules 1–5 using the MRHC paradigm. The algorithm is implemented recursively so that it iterates over all rules as long as new changes are added to the haplotype set. Errors can initially appear after applying rules 3 to 6, and could potentially be propagated by all rules afterwards. If two or more phases at a locus in an individual share the same number of minimum recombinants, that locus is left unphased. A priori, the ideal conditions to reconstruct haplotypes based on the MRHC paradigm should be large full sib families, highly polymorphic loci such as microsatellites, tight linkage and no missing alleles.

This haplotyping approach is deterministic and does not give any estimate of uncertainty associated with the haplotype reconstruction. Hence, we have resorted to simulations to obtain such estimate. Basically, simulated data sets are created conditional on having the same pedigree structure, the same missing data pattern and the same allele frequencies as in the real data. The randomness comes into generating different genotypes among founders and subsequent gene dropping down a pedigree. We will show that, conditional on such restrictions, the proportion of correct phases is almost invariant, and therefore, a single simulation is enough to assess uncertainty of haplotype reconstruction at population level. This is ideal when haplotyping large data sets such as the simulated data in this work, because it drastically reduces computational requirements.

## Methods

Three different data sets were used to test this haplotyping method: wild sheep, farmed pigs and simulated data. Sheep data were collected in St. Kilda, Scotland (54°49'N and 08°34'W). The data consist of a total of 1070 pedigree individuals, approximately 34% of those genotyped at 12 microsatellites scattered over a ~90 cM region on chromosome 11. Approximately 51% of all individuals were fully genotyped at 6 or more loci. The average inter-locus distance and its standard deviation were 7.6 and 7.3 cM, respectively. The number of alleles per locus ranged from 3 to 8, with mode in 3 and 4. The pig data set consisted of a pedigree with 1186 individuals derived from a cross between Meishan and Large-White breeds 7 to 10 generations ago (C. Haley pers. comm.). There were 33 markers, 5 microsatellites and 28 single nucleotide polymorphisms (SNP), scattered over a ~15 cM region on chromosome 7. The average inter-locus distance and its standard deviation were 0.5 and 1.3 cM, respectively. Some loci appeared completely linked, forming 5 multi-locus blocks. They were artificially separated 0.01 cM in order to allow variance components analysis, and the same separation was assumed in this study. Approximately 27.5% of all individuals were fully genotyped at all markers, and 67% were genotyped at 16 markers or more. The simulated data set provided in the XII QTL-MAS workshop consisted of 5939 individuals over 7 generations [2]. All but 74 individuals were genotyped at 1000 SNP loci regularly spaced 0.1 cM apart. These three data sets differ in mating system, family structure, marker information and marker spacing, hence general conclusions about the performance of MRHC can be drawn.

The results presented in this manuscript relate to proportions of alleles phased regarding total number of alleles in full genotypes, as this method cannot phase hemizygous genotypes. Note that we do not give a measure of how many haplotypes are reconstructed without error, as our unit of measurement are alleles within loci rather than haplotypes.

## Results

### Differences in mating system and family size across data sets

Table 1 shows differences in mating system and family size across all data sets with regards to males. For example, the distribution of number of males mated to a given number of different females is L-shaped in sheep and flat in pigs. In contrast, the simulated data set has a simpler mating design in which ~77% of all males mated to 10 different females, producing ~92% of all offspring.

**Table 1:** Mating system and family size from a paternal perspective.

**Sheep**			**Pigs**		
**F**	**M**	**O**	**F**	**M**	**O**

1	252	257	2	3	8
2	24	48	3	2	8
3	4	14	4	3	22
4	4	19	5	3	24
5	1	7	6	3	28
9	1	10	8	2	28
11	5	61	10	5	75
12	1	12	11	2	32
13	2	26	12	4	72
14	1	14	13	5	99
16	2	42	14	3	71
17	3	60	15	2	46
18	1	19	16	1	25
21	1	22	17	2	42
26	2	65	18	2	60
31	1	35	20	1	29
**Simulated**			21	1	33
			
**F**	**M**	**O**	23	1	37
			
8	8	156	24	1	42
9	13	298	25	1	39
10	69	5246	27	2	93

F denotes number of females, M denotes number of males mated to F different females, and O denotes the total number of offspring in those families.

Table 2 shows differences in mating system and family size across all data sets with regards to females. It can be seen that ewes have a not dissimilar distribution to rams (Table 1), i.e. L-shaped, whereas sows are mated at most with four different boars reflecting typical commercial practice. The extreme case of two ewes mated to fifteen different rams can be explained by ewes having twins to different rams (J. Pemberton pers. comm.), and an artificially enriched pedigree after assuming singly missing mates to be unique. All the females in the simulated data set were mated to a unique male, although they could have multiple offspring.

**Table 2:** Mating system and family size from a maternal perspective.

**Sheep**			**Pigs**		
**M**	**F**	**O**	**M**	**F**	**O**

1	184	190	1	236	359
2	51	110	2	94	298
3	22	70	3	41	199
4	11	50	4	9	57
5	2	10	**Simulated**		
			
6	3	18	**M**	**F**	**O**
			
7	1	9	1	871	5700
			
8	4	40			
9	6	64			
10	6	62			
11	3	39			
14	1	15			
15	2	34			

F denotes number of females, M denotes number of males mated to F different females, and O denotes the total number of offspring in those families.

Tables 1 and 2 reflect also sibship size. For example, the average full sib family size in sheep is ~1.1, i.e. the predominant family structure is a trio, whereas the average full sib family size in pigs is ~1.6. In pigs, family sizes were kept artificially constant to ~2 individuals probably due to resource limitations and experimental design requirements, i.e. minimising family variance to simultaneously maximise genetic variability and create balanced designs. The average full sib family size in the simulated data set was ~6.5 offspring.

### Differences in pedigree structure

**Figure 1:**
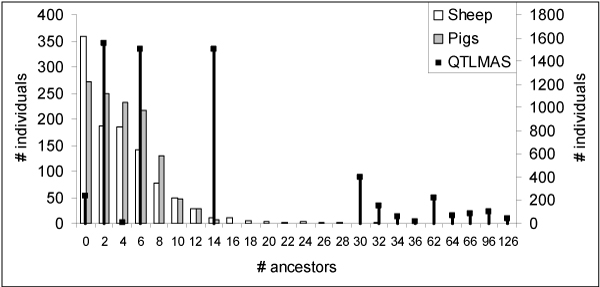
**Pedigree structures**. This figure shows the number of individuals (y-axes) against number of ancestors (x-axis), e.g. there are 359 sheep, 272 pigs and 239 individuals in the QTLMAS data with no ancestors (they are founders). The left y-axis is for sheep (clear bars) and pigs (grey bars), and the right y-axis for QTLMAS data (dark bars with top markers).

Figure 1 shows the three pedigree structures. Pigs have a four generations pedigree, including founders. Eight individuals have 14 ancestors, the maximum found in this data set, which probably consisted of 2 parents, 4 grandparents and 8 great-grandparents. In contrast, the sheep pedigree was deeper with one individual having 32 ancestors, although most individuals (97%) had ≤14 ancestors. The simulated data set had the deepest and most complete pedigree with 7 generations including founders, and a maximum of $\sum_{i=1}^{6}2^{i}=126$ ancestors for any individual, where *i *denotes generation.

### Measuring uncertainty

We simulated 100 times each data set by sampling random genotypes among founders based on observed allele frequencies, followed by gene dropping through the pedigree. The missing data pattern was replicated in each simulation. Each full simulated haplotype set was saved and compared against the MRHC set reconstructed from genotypes and pedigree for all three data sets.

Table 3 shows results of reconstructing haplotypes via MRHC paradigm. Columns 2 to 8 show how different the three data sets were in terms of pedigree size and marker data. Columns 9 to 11 show the average percentage of phased alleles (or loci) given they came from fully genotyped loci, the average percentage of correct phases and the coefficient of variation of correct phases, respectively. The average percentage of correct phases was greater than 99%, and the coefficient of variation of correct phases was lower than 0.001 for all three data sets. Hence, these results suggest that a single simulation would be sufficient to provide a measure of uncertainty for the reconstructed haplotype set as long as features of the data are respected, i.e. pedigree, allele frequencies, missing data pattern. All the simulations were carried out in a Pentium 4 PC with 3.8 Ghz, apart from the simulated data, which took longer to compute. For the simulated data we used the resources provided by the Edinburgh Compute and Data Facilities (ECDF), which is partly supported by the eDIKT initiative [3]. All these results were obtained implementing the first four rules, out of 6, in Qian and Beckmann after some modification (see next section).

**Table 3:** Measuring haplotype reconstruction uncertainty via simulations.

**Data**	**N**	**M**	**SNP**	**Length**	**Av.**	**G**	**1/2 G**	**Phased**	**Correct**	**CV**
Sheep	1070	12	0	90	7.6	34	51	82.5	99.7	0.001
Pigs	1186	33	28	15	0.5	27.5	67	78.1	99.9	0.0003
Sim.	5939	1000	1000	100	0.1	98.75	98.75	94.1	99.2	0.0002

There were 100 simulations per data set. N is the pedigree size. M is the number of markers. SNP is the number of SNP among all markers. Length is the totalcM of the region considered. Av. is the average inter-locus distance incM. G is the percentage of all individuals in pedigree with all loci fully genotyped. 1/2 G is the percentage of all individuals in pedigree with at least 1/2 of loci fully genotyped. Phased is the percentage of phased alleles (or loci) from full genotypes. Correct is the percentage of correctly phased alleles from all phased. CV is the coefficient of variation of correct phases across simulations.

### Implementing partial vs. full MRHC

The MRHC as described in Qian and Beckmann (2002) [1] consists of 6 rules that must be applied sequentially and iteratively until no more alleles can be phased unambiguously. We modified some of the rules in order to improve performance of our software. For example, in figure number 1 of Qian and Beckmann [1], the third locus of individual 6 was phased last by rule 6, however this could have been phased much earlier by rule 4. Thus, we increase the phasing potential of rule 4 by incorporating into it some of the features of rule 6. Table 4 shows results after implementing all or only the first four rules across all data sets. Implementing all rules increased the percentage of phased loci between 2 and 4%, coupled with a slight decrease in the percentage of correct phases between 0.2 and 0.4%, and an increase in computational time between 3 and 5 times. The increase in computational demands can be a problem with large data sets. For example, it took ~1 hour to obtain a haplotype set for the simulated data when using four rules, and ~4 1/2 hours when using six rules. However, despite such increase in computational requirement, only ~2% additional alleles were phased and the percentage of correct phases dropped by ~0.2% compared to just using 4 rules (Table 4).

**Table 4:** Comparison between fully or partially implementing MRHC.

**Data**	Sheep		Pigs		Simulated	
**Rules**	4	6	4	6	4	6
**Phased**	82.5	85.1	78.1	82.3	93.5	95.3
**Correct**	99.7	99.3	99.9	99.6	98.8	98.6
**Time**	1.9	5	5.6	25.2	3460	16339

The original MRHC algorithm contains 6 rules. The first four can be implemented on their own to phase most alleles. Phased was the percentage of phased alleles originally present in full genotypes. Correct is the percentage of correctly phased alleles from all phased. Time measures average computing requirement in seconds. One hundred simulations were used to assess performance in pigs and sheep, and true and predicted haplotypes were compared in the simulated data.

Table 4 was obtained as the average of 100 simulations for the sheep and pig data, and comparing reconstructed haplotypes against true ones for simulated data. The percentage of correct phases when compared reconstructed and true haplotypes was 98.8 when using four rules. This is similar to the 99.2% found through simulations (Table 3).

## Discussion

The MRHC paradigm [1] for reconstructing haplotypes given genotypes and pedigree rendered ~99% of correct phases in three data sets. The percentage of phased alleles varied from ~80 to ~95, and seemed to depend on marker density, marker type, missing data pattern and, possibly, family structure. The MRHC paradigm states that the phase at a locus uniquely leading to the least number of recombinants in a full sib family is preferred over all other alternative phases. Although this methodology may be biased downwards, i.e. it sometimes underestimates the true number of recombinations, our results show that this bias must be very small.

There was almost no variation in the percentage of correctly phased alleles across simulations based on features of the original data (CV<0.001). This low CV was observed across three different data sets: ~1000 sheep with a dozen microsatellites over 100 cM and small full sib families, ~1000 pigs with 33 loci (SNPs and microsatellites) over 15 cM and slightly larger full sib families, and ~6000 individuals with 1000 SNPs over 100 cM with relatively large full sib families. If this was general, as it seems to be, then it would enable us to associate a population-wide uncertainty estimate to any reconstructed haplotype set based on a single simulation.

This finding was corroborated by haplotyping the simulated data, for which true haplotypes were known. The total proportion of phased alleles present in full genotypes varied more than the proportion of correctly phased alleles from all phased alleles, e.g. the CV of both proportions were ~0.01 and ~0.001, respectively, in the sheep. This indicates this method is conservative as it rarely tries to phase alleles unless certainty is high. Any improvement of this method must be in the direction of increasing the proportion of phased alleles without increasing the error rate.

Haplotype frequencies obtained under the MRHC paradigm could be fed as priors to haplotype unrelated individuals using maximum likelihood [4].

The version of the MRHC paradigm implemented in this work has been incorporated into the Linkage Disequilibrium and Linkage Analysis (LDLA) module within the GridQTL software [5].

## Conclusion

Given that haplotypes are essential to estimate linkage disequilibrium and can improve power of gene detection, reliable and efficient methods to estimate them are required. Being a deterministic method, it is reasonably fast, taking from seconds to haplotype ~500 individuals at 10–30 loci, and from 1 to 5 hours to haplotype ~6000 individuals for 1000 loci. It is also highly accurate, reaching ~99% of correct phases in three very different data sets.

## Competing interests

The authors declare that they have no competing interests.

## Authors' contributions

JHS implemented MRHC in Fortran, carried out the simulation study, and wrote the original draft manuscript. SK participated in the study design and coordination and helped to draft the manuscript. All authors read and approved the final manuscript.
